# Hippocampal circuit dysfunction in psychosis

**DOI:** 10.1038/s41398-022-02115-5

**Published:** 2022-08-25

**Authors:** Samuel Knight, Robert McCutcheon, Daniella Dwir, Anthony A. Grace, Owen O’Daly, Philip McGuire, Gemma Modinos

**Affiliations:** 1 Department of Psychosis Studies, Institute of Psychiatry, Psychology & Neuroscience, King’s College London, London, UK; 2 Center for Psychiatric Neuroscience, Department of Psychiatry, Lausanne University Hospital (CHUV), Lausanne, Switzerland; 3 Departments of Neuroscience, Psychiatry and Psychology, University of Pittsburgh, Pittsburgh, PA USA; 4 Department of Neuroimaging, Institute of Psychiatry, Psychology and Neuroscience, King’s College London, London, UK; 5 NIHR Maudsley Biomedical Research Centre, London, UK; 6 MRC Centre for Neurodevelopmental Disorders, King’s College London, London, UK

**Keywords:** Schizophrenia, Biomarkers, Clinical pharmacology

## Abstract

Despite strong evidence of the neurodevelopmental origins of psychosis, current pharmacological treatment is not usually initiated until after a clinical diagnosis is made, and is focussed on antagonising striatal dopamine receptors. These drugs are only partially effective, have serious side effects, fail to alleviate the negative and cognitive symptoms of the disorder, and are not useful as a preventive treatment. In recent years, attention has turned to upstream brain regions that regulate striatal dopamine function, such as the hippocampus. This review draws together these recent data to discuss why the hippocampus may be especially vulnerable in the pathophysiology of psychosis. First, we describe the neurodevelopmental trajectory of the hippocampus and its susceptibility to dysfunction, exploring this region’s proneness to structural and functional imbalances, metabolic pressures, and oxidative stress. We then examine mechanisms of hippocampal dysfunction in psychosis and in individuals at high-risk for psychosis and discuss how and when hippocampal abnormalities may be targeted in these groups. We conclude with future directions for prospective studies to unlock the discovery of novel therapeutic strategies targeting hippocampal circuit imbalances to prevent or delay the onset of psychosis.

## Introduction

Hippocampal dysfunction is a robust feature in the pathophysiology of psychosis [1, 2]. In patients with a psychotic disorder such as schizophrenia, hippocampal volume is reduced [3–5] and function is abnormal: neuroimaging measures of resting activity including positron emission tomography (PET) [6], resting-state functional MRI (rs-fMRI) [7–10], and resting cerebral blood volume (CBV) [11–13], converge to suggest that the hippocampus is hyperactive in psychosis. Moreover, hippocampal alterations are already present before illness onset in people at clinical high-risk (CHR) of psychosis, including increased hippocampal resting cerebral blood flow (rCBF) [14, 15] and disrupted hippocampal-basal ganglia and hippocampal-prefrontal connectivity [16–18]. These human findings are broadly consistent with preclinical data demonstrating that hippocampal hyperactivity drives the development of striatal dopamine dysfunction [19] and associated psychosis-relevant behaviours [20]. If hippocampal dysfunction plays such a key role in the onset of psychosis, correcting this dysfunction may represent a promising strategy for the development of new treatments and preventive interventions. The aim of the present review is to explore recent developments in the study of hippocampal circuit dysfunction in psychosis, understand what they mean in the context of normal hippocampal neurodevelopment or under exposure to known environmental risk factors for psychosis, and how these findings can inform the development of novel treatments.

## Hippocampal circuitry

**Fig. 1:**
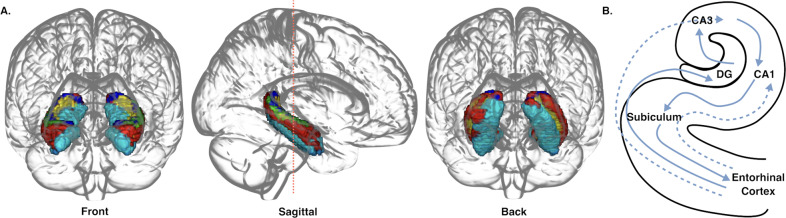
Hippocampal subfield anatomy. **A** The subdivisions of the hippocampus along its long axis—the dentate gyrus (DG) and four sections of the cornu ammonis (CA1 red; CA2, blue; CA3, green; CA4/DG, yellow; subiculum, cyan)—are demarcated early in prenatal neurodevelopment through specific genetic molecular markers. Segmentation derived from cytoarchitectonic anatomical probability map [244]. **B** Cross-section of left hippocampi, highlighting primary internal circuitry of the hippocampal formation. Solid lines reflect the ‘trisynaptic circuit’, dash lines reflect supporting entorhinal circuitry. Each subdivision of the hippocampus is linked to the neighbouring entorhinal cortex through the ‘trisynaptic circuit’, an excitatory projection that links hippocampal subregions via the perforant pathway to granule cells in the DG. These granule cells are linked to pyramidal cells in region CA3 via mossy fibres, which in turn project to pyramidal cells in CA1 via Schaffer collaterals, before exiting the hippocampus via the subiculum [245]. In addition to the trisynaptic circuit, there are supporting connections between the entorhinal cortex and CA1 and CA3, and subiculum, as well as projections between CA1 and CA3. The hippocampus receives input primarily from the entorhinal cortex but is also extensively connected with proximate regions, including the anterior cingulate (ACC), medial prefrontal cortex (mPFC), and amygdala [246]. The hippocampus sends direct outputs to the nucleus accumbens, hypothalamus, and thalamus, and indirect outputs to the striatum via the nucleus accumbens and ventral tegmental area [247].

The hippocampal formation comprises several distinct, densely packed, highly connected areas (Fig. 1). The circuitry of the hippocampus is inextricably linked to its functions—pattern completion and separation—which requires distinct subfield contributions [2]. Hippocampal cells also recruit distal cortical regions in hippocampal-cortical circuits, processing information involving memory, spatial navigation, emotion, and stress [21].

Together, these hippocampal-cortical circuits form a systematic mental representation of accumulated knowledge and experiences, which can be conceptualised as a ‘cognitive map’ [22]. When a neuronal assembly relating to a particular cognitive map is cued, this triggers a sequence of oscillatory signalling which preferentially follows an encoded representation, thereby supporting the prediction of event consequences and prompting behaviour. These patterns are believed to be consolidated and integrated during rest, when hippocampal sharp-wave ripple oscillations reactivate the sequence of neural assemblies in the absence of the original stimuli—a process known as neural replay [23]. The circuitry of hippocampal subregions is crucial for processes used to distinguish between (pattern separation) and link (pattern completion) cognitive maps [2]. Cognitive maps are a useful framework for understanding psychotic symptoms. If the dynamics cueing neurons and attracting signal through a cognitive map become imbalanced, pattern separation and completion processes are compromised [2]. The resulting “shallow cognitive maps”—over-general pattern completion and reduced pattern separation—could give rise to the cognitive impairments, thought disorder, and aberrant salience in psychosis through the reinforcement of loosely associated circuits during neurodevelopment (see [24] for a review).

## Neurodevelopment of hippocampus

The internal circuitry of the hippocampus develops in a complex and lengthy process. Neural cell proliferation, migration, differentiation, and synaptogenesis all begin prenatally and extend through the first years of postnatal life, with synaptogenesis continuing right through to adolescence [25]. The developmental timeframe of these processes reflects critical or sensitive periods of brain maturation—developmental windows where brain circuitry is shaped by postnatal sensory stimulation—that are essential for healthy neurodevelopment [26].

During early life, sensory information in the environment influences the organisation of the brain through effects on synaptogenesis, pruning, and myelination. Development is rapid through the first year of postnatal life; most structures are already established, and the pace of subsequent synaptogenesis slows [27]. A critical period for synaptogenesis is estimated to close in early childhood [28, 29], as the mental representations of early life experiences become encoded and distributed in neural circuits. This leads to another critical period in human development in adolescence, with synaptic pruning and myelination processes accelerating to solidify these neural patterns, before tapering off in early adulthood [25].

Specific maturational processes are difficult to characterise in vivo [30], although these are indirectly reflected through MRI-derived grey matter volume decreases and white matter volume increases between childhood and early adulthood [31]. Unlike much of the cortex, several subcortical structures including the hippocampus display a contrasting lack of hippocampal grey matter decline over adolescence and early adulthood [32–34], suggesting that the processes involved in synaptic pruning over neurodevelopment may be occurring over a protracted period and potentially leaving the hippocampus more vulnerable to aberrant adolescence development. Unlike other structures, there is also ongoing postnatal neurogenesis within the dentate gyrus (DG), likely contributing to protracted hippocampal development. The rate of neurogenesis appears to taper off in early adulthood [35], though there is other evidence that it persists into adulthood [36]. Overall, the role of ongoing neurogenesis is undoubtedly important for hippocampal development and the emergence of psychotic phenotypes (see [37–39] for reviews).

Developmental changes within the hippocampus are also not uniform. The posterior/dorsal hippocampus, most often associated with memory and spatial learning, gains in volume as a ratio to its size at age four, over 19 years of subsequent development [40]. Conversely, the anterior/ventral hippocampus, with projections to the prefrontal cortex and amygdala and associated with socio-emotional and stress response processes, decreases in relative volume over the same period. The differences in anterior/posterior specialisation and development are relevant for psychosis, as socio-emotional and stress response abnormalities in psychosis [41] are particularly accentuated in adolescence [42].

This vulnerability of the hippocampus may particularly impact normal development by its dense connectivity. The hippocampus is part of the so-called “rich club” of hub brain regions (including the superior frontal cortex, precentral gyri, thalamus, and putamen), with particularly high network centrality and vulnerability to dysfunction [43, 44]. There is diverse connectivity between hippocampal subfields and known resting-state networks in mature brains [45]; these networks are already partially established by age four and continue to consolidate over childhood [46]. Together, the protracted neurodevelopment and high connectivity of the hippocampus may leave immature neurons vulnerable to damage during a period where risk factors for psychosis may be particularly influential.

## Hippocampal alterations in psychosis

Hippocampal volume deficits are evident at the first episode of a psychotic disorder (FEP), when the clinical diagnosis is first made, and are most marked in anterior cornu ammonis (CA) regions [47]. These reductions also appear to be greater in patients with relatively severe positive symptoms [48], as well as in those in whom there was a long delay before treatment was initiated [49], though hippocampal volume reductions do not appear to be progressive from FEP to chronic illness [47, 50, 51].

Abnormal metabolic activity within the hippocampus may precede and drive hippocampal volume loss [1]. Elevated CBV left anterior CA1 was found in CHR individuals who subsequently transitioned to psychosis [52]. After a 24-month follow-up, higher CBV was also evident in the subiculum in the CHR subgroup that transitioned to psychosis, overlapping with longitudinal MRI volume loss. Parallel animal model work involving the chronic administration of ketamine produced a similar pattern of hippocampal CBV increase and volume reduction, suggesting that these MRI-based changes may be driven by local glutamatergic dysfunction [52]. In humans, longitudinal neuroimaging data suggest that increased hippocampal rCBF normalises in CHR individuals who subsequently remit from the CHR state [15], whereas in CHR subjects with adverse clinical outcomes, elevated hippocampal rCBF has been associated with lower striatal dopamine synthesis capacity [53]. Small hippocampal volume reductions been reported in people at CHR for psychosis [54, 55], which may be more prominent in CA1 [56]. There is also evidence that these reductions at baseline are more pronounced in the subgroup of CHR subjects go on to develop psychosis, and that they continue to decrease longitudinally as these individuals transition to psychosis [57, 58]. However, a recent meta-analysis suggests these findings have not been consistently replicated [59]. In the most recent structural MRI study to date in CHR individuals, baseline grey matter volume in the hippocampus or other cortical regions was not predictive of remission or transition to psychosis [60]. Longitudinal studies in an early psychosis or CHR population are therefore necessary to expand on the links between increased hippocampal metabolism, glutamate dysfunction, and subsequent volume loss.

Alterations in hippocampal structure and function have also been associated with psychotic-like experiences in the general population [61–63] (although see [64]). Moreover, hippocampal dysfunction is shared across other neuropsychiatric disorders [65, 66], which is perhaps unsurprising given the large overlap in genetic risk and symptoms [67]. Due in part to this overlap, hippocampal circuit dysfunction specific to psychosis is often difficult to discriminate [6, 68]. Notwithstanding, there is some evidence that hippocampal alterations are most pronounced in schizophrenia, decreasing in severity along the psychiatric spectrum [4, 69, 70]. The nature and specificity of hippocampal dysfunction in psychosis is thus still being parsed, and while beyond the scope of the present review, this overlap has broad transdiagnostic implications for the pathophysiology and putative treatment of other disorders.

## Causes of hippocampal alterations in psychosis

### Excitation–inhibition imbalance

**Fig. 2:**
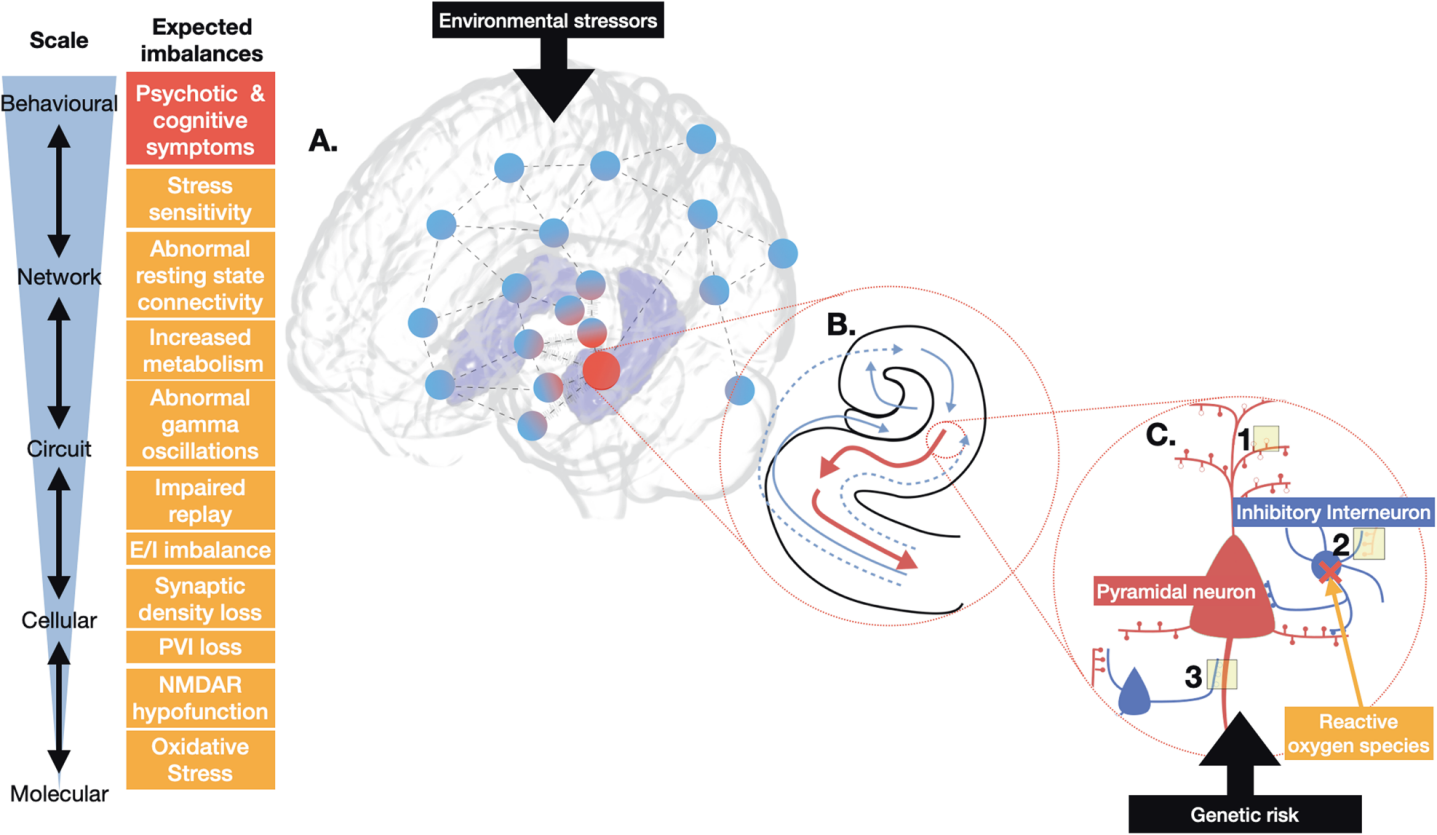
Model of hippocampal hyperactivity in early psychosis, highlighting potential targets for detection/intervention. **A** Stressors lead to cascade of down-scale imbalances, including increased hippocampal metabolism and network (blue spheres) dysfunction (red spheres). Hub regions, including the hippocampus, are most likely to be impacted [43]. **B** Excitatory/inhibitory imbalance and hyperactivity in hippocampal subregions (enlarged arrows). **C** Oxidative stress damages metabolically demanding parvalbumin-positive interneurons (PVI), resulting in N-methyl-D-aspartate receptor (NMDAR) hypofunction and (1) altered pyramidal signalling; (2) reduced pyramidal input to interneurons; (3) reduced interneuron inhibition of pyramidal cells, and a cascade of up-scale imbalances These processes are accentuated in those at highest genetic risk. Diagram adapted in part, with permission [248].

The balanced interplay between excitatory and inhibitory (E/I) neural activity is crucial for regulating brain excitability and the synchronisation of signalling between disparate cortical regions involved in cognitive functioning [71]. Psychosis may involve an imbalance between E/I signalling (Fig. 2), proposed to drive hippocampal hyperactivity and “shallow cognitive maps”, resulting in aberrant coupling between loosely associated neural assemblies [24]. One example of this is impaired hippocampal neural replay in schizophrenia, where abnormal hippocampal oscillations impair learning [72].

In most cortical regions, ~80% of neurons are excitatory glutamatergic pyramidal cells, whereas in the hippocampus these cells represent ~90% of all neurons [73]. Because only ≈10% of hippocampal neurons are inhibitory GABAergic interneurons, this region may be more susceptible to E/I imbalance. For example, the hippocampus is a source of high-frequency gamma oscillations (~30–100 Hz range) that are implicated in memory encoding, storage, and retrieval [74, 75], and known to be disrupted in psychosis [76]. Gamma oscillations are regulated by fast-spiking GABAergic parvalbumin-positive interneurons (PVI) [77, 78]. In transgenic mice, optogenetic stimulation of PVI generates gamma oscillations and improves cognitive performance [79], while disruptions to N-methyl-D-aspartate receptor (NMDAR) input on PVI impair gamma oscillations [80]. PVI play a role in hippocampal neurogenesis [81], and constitute around 20% of hippocampal inhibitory neurons, somewhat more concentrated in CA1 and CA3 than in the DG [82]. This compares to around 40% of cortical interneurons [83].

In psychosis, reductions in hippocampal PVI density have been found in *post-mortem* studies [84, 85] and hippocampal PVI cell loss is evident in several animal models of psychosis [52, 78]. There is also significant *post-mortem* and in vivo evidence of synaptic deficits in psychotic disorders [86–89], which may be a result of PVI impairments and may drive hippocampal hyperactivity. PVI synaptogenesis is regulated by Neuregulin 1 and Erbb4 receptors, which are also implicated in neurodevelopmental and psychotic disorders [90]. Experiments in a genetic animal model involving Erbb4 deletion from PVI show lower synaptic density as indexed by synaptic vesicle glycoprotein 2A (SV2A) sampling, together with increased rCBF in the ventral hippocampus [91]. These results resemble neuroimaging findings associated with psychosis-risk [14, 86], linking PVI and SV2A deficits with hippocampal hyperactivity.

PVI dysfunction has also been linked to NMDAR hypofunction in psychosis [92, 93]. NMDAR antagonists such as phencyclidine decrease PVI count [94], thereby leading to lower PVI inhibition of pyramidal neurons. Moreover, activation of the NR2A subunit of NDMARs contributes to the maturation of PVI [95] and therefore NMDAR hypofunction may be most detrimental to PVI early in development [96].

Single-photon emission computed tomography (SPECT) and PET have been used to quantify NMDAR density in vivo in patients with psychosis. While the specificity of these radioligands for the receptor remains controversial, one SPECT study reported reduced NMDAR binding in the left hippocampus relative to the whole cortex in unmedicated patients with psychosis, but this deficit was ameliorated in antipsychotic treated patients [97]. A recent PET study replicated this finding in early psychosis; FEP patients showed lower hippocampal NMDAR binding relative to the whole cortex compared to healthy volunteers, although no differences in hippocampal NMDAR availability were observed [98]. Overall, there is some evidence that hippocampal abnormalities in psychosis may be related to NMDAR hypofunction.

Although currently PVI activity cannot be measured in vivo in humans, levels of glutamate and GABA partially reflect E/I processes [99] and can be measured with proton magnetic resonance spectroscopy (^1^H-MRS). Meta-analysis of ^1^H-MRS studies in patients with schizophrenia, FEP, and CHR individuals showed elevations in levels of glutamate + glutamine (Glx) in the basal ganglia of schizophrenia and FEP patients, regardless of whether they were medicated [100]. However, hippocampal Glx was only elevated in unmedicated patients, with no elevations in basal ganglia Glx in CHR unless combined with patient groups. In CHR individuals, one study found higher levels of hippocampal glutamate levels in the CHR subgroup that subsequently transitioned to psychosis[101], although another study did not [102]. One multimodal neuroimaging study found that hippocampal glutamate levels and striatal dopamine synthesis capacity were negatively correlated in CHR individuals who later developed psychosis [103]. This is an intriguing finding given the proposed causal link between hippocampal hyperactivity and elevated striatal dopamine [104], though important to note that Glx signal acquired through ^1^H-MRS captures more than hippocampal-striatal projections and includes inputs to the anterior hippocampus. If inhibitory signalling is perturbed, one would predict a Glx decrease.

In terms of GABA levels, relatively few studies have investigated these in the hippocampus in patients with psychosis, partly due to the technical difficulties in reliably positioning a GABA-edited ^1^H-MRS voxel in this region [105]. Nevertheless, ^1^H-MRS studies at both 3T and 7T have not found significant alterations in absolute concentrations of GABA in patients with psychosis compared to healthy controls [106, 107]. In a multimodal imaging study in people at CHR, GABA levels in the prefrontal cortex were correlated with hippocampal rCBF, a correlation driven by those individuals who subsequently transitioned to psychosis [108]. Some of the technical difficulties in measuring hippocampal GABA in vivo with ^1^H-MRS may be overcome by using PET imaging. A recent study reported lower GABA_A_ α5 receptor availability (highly expressed in hippocampus) only in the subgroup of patients with schizophrenia that were antipsychotic-free [109], suggesting that PET measures of GABA dysfunction can be influenced by the effects of antipsychotic medications [110].

### Stress sensitivity

Environmental stressors (including poverty, ethnicity/immigration, and childhood adversity) are among the strongest risk factors for psychosis [111]. The hippocampus appears to play a role in mediating their effects; preclinical studies have found increased hippocampal activity and PVI loss following environmental stressors (including maternal separation and random foot shocks) [112, 113], particularly before adulthood [114]. In children, environmental stressors such as maltreatment, poverty and neglect have deleterious effects on anterior hippocampal volume [115]. While hippocampal volume loss may not occur after a single traumatic event [116], the extended period of vulnerability allows for an accumulating cascade of maladaptive environmental influences on neurodevelopment [117]. In CHR individuals, a history of childhood trauma has been associated with reductions in hippocampal volume in early adulthood [14]. The impact of early life risk factors may vary according to sex: females may be more resilient to certain types of early maltreatment [118], but more susceptible to abuse later in life [119]. These differences in vulnerability to stressors may contribute to the later age of onset of psychosis in females [120], and raises the possibility of sex-dependent differences on the putative window for effective preventive intervention.

A possible mechanism by which early life stressors may precipitate metabolic pressures on the hippocampus is through its involvement in stress and emotion processing [121]. The hypothalamic pituitary adrenal (HPA)-axis regulates the response to stress through cortisol binding to glucocorticoid and mineralocorticoid receptors, which are present at a high density in the anterior hippocampus [122]. While this stress response is critical for normal learning [123], repeated activity burdens the system [124]. Hence, early life stressors may alter hippocampal neuronal structure [125] and potentially sensitise the HPA-axis. Environmental stressors during key developmental periods may reinforce a stress-sensitised phenotype, with aberrant synaptic pruning in adolescence leading to altered E/I balance and dysfunctional cognitive maps, restricting the deployment of alternative cognitive strategies. For instance, excess glucocorticoid activity reduces the complexity of CA3 hippocampal pyramidal cells [126] and inhibits neurogenesis [127]. The effects of these stressors on the HPA-axis may be especially pronounced in adolescence due to neurohormonal processes involved in puberty [128]. Sustained activation of glucocorticoids during vulnerable developmental periods may also impair energy metabolism in the hippocampus and lead to oxidative stress [129].

### Oxidative stress

Oxidative stress results from an impairment in the redox cycle of oxygen metabolism. Redox dysregulation occurs when there is an imbalance between reactive nitrogen and oxygen species (ROS), and the capacity of the cells to detoxify the ROS with antioxidant defences, predominantly through the reduction of glutathione (GSH) [130]. ROS are deleterious by-products of aerobic metabolism, resulting from the mitochondrial electron transfer chain that converts glucose and oxygen into adenosine triphosphate [131]. High-energy demands can perturb the redox balance and result in ROS-mediated damage of surrounding molecules [130]. The hippocampal region may be particularly vulnerable to oxidative stress because it is a metabolically active region, with high degree of connectivity to distal regions and relatively fine-tuned excitatory/inhibitory cell balance.

PVIs display a high rate of firing relative to other neurons, and therefore have relatively high metabolic demands [132]. The generation of gamma oscillations, mediated by hippocampal PVIs, can require more than double the baseline oxygen consumption, making PVIs particularly vulnerable to oxidative stress [133]. Glutamate cysteine ligase (GCL) is the rate-limiting enzyme for synthesising GSH, and a GCL modulatory subunit (M) knockout model shows depleted GSH [134] and loss of PVI [135]. Indeed, oxidative stress has been shown to disrupt gamma oscillations from ventral CA3 in a mouse model of relevance to psychosis [134]. Oxidative stress is a vicious cycle whereby ROS, if not neutralised, may impair mitochondrial function, generating even more ROS.

Converging evidence links psychosis with increased oxidative stress [136–138]. Schizophrenia patients have increased DNA oxidation, lipid oxidation/peroxidation, and free radicals [138], as well as decreased antioxidants [137]. A meta-analysis of oxidative stress in schizophrenia found differences in 10 peripheral oxidative stress markers—from red blood cells, plasma, and serum—depending on FEP or chronic schizophrenia clinical status [137]. Though most studies focus on peripheral markers of oxidative stress, oxidative DNA damage in hippocampal tissue was found to be 10× higher in patients with chronic schizophrenia than in non-psychiatric subjects by *post-mortem* examination [139].

Oxidative stress is difficult to spatially localise in vivo, so instead researchers look for signs of redox dysregulation, with GSH levels as a widely used marker of an impaired antioxidant system. Animal model evidence for an association of reduced GSH levels with hippocampal abnormalities [130] implicates oxidative stress in the pathophysiology of psychosis. For instance, GSH-deficient transgenic mice show a time-dependent impairment of PVI functioning in the ventral hippocampus, linking GSH to PVI damage/loss [134]. In this same mouse model, reduced fractional anisotropy (FA) and conduction velocity of slow-conducting fibres of the fornix-fimbria were found during adolescence [140], suggesting connectivity impairments of the hippocampus to other subcortical areas. Interestingly, reduced FA in the fornix is also observed in patients at the early stage of psychosis, underscoring the potential role of oxidative stress in these alterations [141].

In humans, recent meta-analyses of ^1^H-MRS studies found a persistent pattern of GSH deficits in psychosis [142, 143], with most studies focusing on the mPFC/ACC. Hippocampal GSH has not been studied extensively in psychosis, owing to similar technical limitations to the use of ^1^H-MRS for measuring hippocampal GABA levels, although one study found higher GSH in the medial temporal lobe of FEP patients versus healthy controls [144]. In this context, peripherally measured redox blood markers and GCLC genotyping may be more practical markers of redox status and dysfunction than brain GSH measures. A genetic polymorphism in the catalytic subunit of the GCL was found to be associated with schizophrenia and led to decreased brain [145] and blood GSH levels, as well as GCL activity [146]. One study found that mPFC GSH became uncoupled from peripheral GSH-related enzymes in early psychosis patients, but remained positively correlated in healthy participants [145], whilst another linked higher peripheral glutathione peroxidase with reduced hippocampal volume in early psychosis but not in healthy participants [147]. Moreover, this association was also found in patients that had experienced childhood trauma, linking adverse life events with increased oxidative stress and hippocampal alterations [148]. Though this evidence supports a role for redox dysregulation in psychosis, further studies are warranted to determine hippocampal GSH levels in the disorder, and the links between peripheral redox markers and hippocampal activity.

The effects of oxidative stress in relation to psychosis may be especially important in preadolescence, when they may affect perineuronal nets (PNN)—an extracellular matrix of molecules that enclose neurons and provide them with structural support [133]. Their presence around PVI is essential in closing a period of plasticity in preadolescence in which synaptic pruning and apoptosis occur [26, 149, 150]. PNN also protect PVI from oxidative stress, although they themselves are still vulnerable to oxidative damage [151]. *Post-mortem* analyses have found a 70% reduction of PNN in patients with psychosis in the PFC [152], however, evidence of PNN deficits in the human hippocampus is extremely limited [153, 154]. Clearly, further characterisation of PNN in the human hippocampus is required. However, a study of the methylazoxymethanol acetate (MAM) preclinical model for psychosis found a reduction in PNN, which was linked to increased firing of hippocampal pyramidal cells [155]. Furthermore, chronic stress in adolescence—but not adulthood—reduces PVI and PNN [114]. Collectively, these observations suggest that oxidative stress may damage hippocampal PVI and PNN, extending a neurodevelopmental period when there is scope for aberrant plasticity and synaptic pruning [149].

## Targeting hippocampal dysfunction in psychosis

**Fig. 3:**
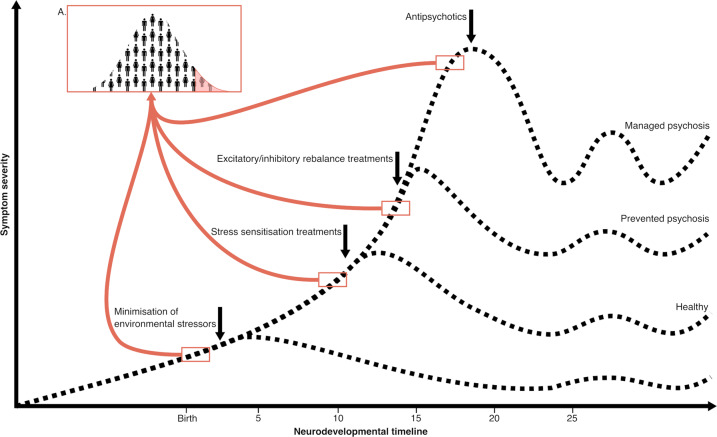
Prototypical psychosis developmental timeline with suggested periods for targeted interventions to alter psychosis trajectory. Individuals at higher risk could be identified through normative modelling [232] at different neurodevelopmental timepoints (**A**). Modelling may include a regional vulnerability index [50] or other hippocampal markers (Table 1). Personalised treatments could then be tailored to specific anomalies.

This section will consider how the aforementioned hippocampal circuit abnormalities may be targeted during neurodevelopment to reduce the cascade to a psychotic disorder (Fig. 3).

### E/I imbalance

One method for correcting hippocampal hyperactivity may be through targeting glutamate. For example, in a mouse model of psychosis, an mGluR 2/3 agonist prevented ketamine-induced hyper-metabolism of the hippocampus [52]. Furthermore, peri-pubertally targeting this receptor with the mGluR 2/3 agonist pomaglumetad methionil normalised VTA dopamine and ventral hippocampal pyramidal neuron activity in the MAM model of psychosis [156]. However, to date, results from clinical trials of compounds that target glutamate function in patients with psychosis have been disappointing. Pomaglumetad methionil was inferior to aripiprazole in relieving positive symptoms [157], and no better than placebo as an adjunctive treatment for negative symptoms [158]. Bitopertin, a glycine transport inhibitor, was ineffective at improving symptoms in schizophrenia in phase-III trials [159]. Still, these studies involved patients with a chronic psychotic disorder, who had been treated with antipsychotic medications. It is possible that glutamatergic medications might be more useful if given in the early phase of psychosis, in FEP or CHR individuals, before receiving prolonged antipsychotic treatment.

Another strategy to redress E/I imbalance is to target the GABAergic system to restore inhibition within the hippocampus. For instance, in the MAM model for psychosis, the experimental transplantation of GABAergic precursor cells into the ventral hippocampus normalised hippocampal and striatal dopaminergic function [160]. Similarly, in the same model, peri-pubertal administration of diazepam prevented PVI loss and hyperdopaminergia at adulthood [161]. To date, experimental interventions with GABA-enhancing medications have mainly involved benzodiazepines, which are broad GABA_A_ agonists [162] and there is no evidence for antipsychotic efficacy of additional benzodiazepine medication in schizophrenia [163]. Levetiracetam is an antiepileptic drug that binds to SV2A [164], consequently enhancing GABAergic signalling [165] and regulating E/I balance [166]. Several clinical trials are currently ongoing with levetiracetam in schizophrenia: NCT04317807, NCT03129360, NCT03034356, and NCT02647437. As with novel glutamatergic compounds, these trials are being conducted in patients with well-established psychosis, rather than patients in its early phase, prior to antipsychotic use. This may reduce the chances of detecting clinical effects, as antipsychotic medications can confound or block the effects of GABAergic intervention [109, 167]. Of greater concern are case reports suggesting that levetiracetam can induce psychosis in patients with epilepsy, particularly in patients with a history of psychosis, so may be harmful in some patients [168, 169]. The recent advent of more specific GABA_A_ α5 receptor subunit modulators are of great interest, due to their relative specificity for the hippocampus and their effectiveness in animal models [170].

Sodium Valproate is another anticonvulsant drug often used to treat mania [171] which has also been used effectively to augment antipsychotic treatment [172]. Valproate’s acute benefits for mania and epileptic seizures are likely partially due to an increase of endogenous GABA levels, but valproate also inhibits histone deacetylation and may have enduring effects on gene expression [173]. Interestingly, valproate may be able to reopen certain critical periods for learning [114, 174]. This raises the possibility that valproate could be used to retrain maladaptive cognitive maps and perhaps extend the neurodevelopmental window prior to psychosis onset to allow PNN to properly develop. However, extending these critical periods without sufficient support may leave patients vulnerable to the aforementioned metabolic pressures [149] and stressors [114].

### Oxidative stress

N-acetyl cysteine (NAC) is an antioxidant and a precursor to GSH. Its administration has been shown to instigate a 23% increase in mPFC GSH, improvement of cognitive symptoms and increase in white matter integrity in the fornix [175] of early psychosis patients [176]. Administration of NAC to juvenile and adolescent rats with neonatal hippocampal lesion—a well-established model for psychosis—rescued the development of behavioural phenotypes associated with psychosis in adulthood [177]. NAC also rescues the PVI/PNN maturation impairments found in GSH-deficient GCLM knockout mice [135, 178, 179]. Collectively, this preclinical evidence indicates that NAC has potential as a treatment for psychosis.

A meta-analysis of 7 studies found that in patients with psychosis, NAC improved both positive and negative symptoms after 24 weeks of treatment [180]. However, in theory, NAC could be even more useful if administered prior to the onset of psychosis. If oxidative stress damages PNN/PVI in adolescence, before they are mature, it is crucial that strategies tailored to oxidative stress are developed to target these developmental periods. In preclinical studies, NAC has been administered to juvenile and peri-pubertal animals, during PVI/PNN development. In humans, the earliest that NAC has been given is after the first episode of psychosis [180], though a clinical trial in CHR subjects is currently ongoing [181].

In addition to NAC, other food supplements may reduce the effects of oxidative stress. Sulforaphane is an extract from broccoli that increases peripheral and hippocampal GSH in healthy volunteers [182], and is being evaluated in CHR subjects [183]. Nicotinamide mononucleotide is important in the biosynthesis of nicotinamide adenine dinucleotide, which itself plays an important role in energy metabolism [184] and has reduced levels in patients experiencing FEP and their non-psychotic siblings [185], warranting further investigation. Resveratrol, another antioxidant, is promising for its neuroprotective effects in animal model studies but so far has had little success in human trials [186]. Dietary supplement of ω-3 polyunsaturated fatty acids, antioxidants that reduce inflammation, may also be beneficial [187, 188]. However, despite initial success [189], the most extensive clinical trial to date failed to replicate ω-3 reducing CHR transition to psychosis [190]. Future studies investigating antioxidant supplementation may consider including peripheral markers of redox status, to identify whether these interventions may be helpful for a subset of patients with the most compromised antioxidant defences.

### Cannabidiol (CBD)

Cannabis use is a robust risk factor for psychosis [191], and this effect is attributable to its constituent delta-9-tetrahydrocannabinol (THC). However, another of its constituents, cannabidiol (CBD), is anxiolytic [192], neuroprotective [193], and appears to have antipsychotic effects [194]. CBD’s precise mechanism of action is unclear but appears to be different from that of antipsychotic medications and other potential treatments for psychosis. One plausible mechanism is through cannabinoid (CB) receptors, where CBD acts as a negative allosteric modulator and antagonises CB1&2 agonists [195], possibly in opposition to THC which is a CB1 receptor agonist [196]. The hippocampus is one of the most densely populated regions with CB1 receptors, along with the frontal cortex and basal ganglia [197]. Likewise, CB2 receptors, once only thought to be expressed peripherally, have now been found in the hippocampus and midbrain, though in much lower concentrations than CB1 [198]. CB1 activation disinhibits pyramidal cells and promotes hippocampal hyperactivity [199], as well as reducing theta, gamma, and ripple oscillations in the hippocampus [200], conceivably contributing to the detrimental effects of THC in psychosis. Conversely, CB2 receptors may function in opposition to CB1 [201]. By preventing endogenous CB1 receptor agonism and activating CB2, CBD may assist in stabilising this pathway. In support of this view, CBD increases rCBF in the hippocampus, but not in the amygdala, orbitofrontal or prefrontal cortices [202]. SPECT studies of CBD also demonstrate some hippocampal specificity: CBD reduced rCBF in the hippocampus, parahippocampal and inferior temporal gyri, and increased rCBF in the posterior cingulate gyrus [203, 204]. Several other mechanisms of CBD have been proposed including regulating the GPA axis through facilitating 5-HT1A neurotransmission [205] and inhibition of fatty acid amide hydrolase [206]. Other putative targets identified pre-clinically include GPR55 and transient receptor potential vanilloid type 1 [207, 208], though further work is needed to understand the role of these receptors in psychosis.

Preliminary work using CBD as a treatment of psychosis indicates that it is effective in reducing psychotic symptoms, both alone and when used as an adjunct to antipsychotic medications [194, 209]. In CHR individuals, a single dose of 600 mg CBD normalised brain activation in regions that showed abnormal responses under placebo, including the hippocampus [210]. One week of treatment of 600 mg/day CBD significantly reduced cortisol reactivity in healthy control participants compared with a placebo-administered CHR group, while the CBD-administered CHR group experienced an intermediate but non-significant reduction in cortisol reactivity [211], which may support its use as a stress-desensitisation treatment. Longitudinal studies in CHR individuals are needed to ascertain whether CBD reduces symptoms or likelihood of transition to psychosis.

### Behavioural interventions

In theory, stress sensitivity could be targeted through interventions that reduce the risk of exposure to early life stressors, or that provide individuals with strategies to cope with stressors when they are active. Environmental enrichment prevented hippocampal hyperactivity in the MAM model for psychosis [212], and may be particularly effective when combined following NAC treatment [178]. In a longitudinal community sample, an environmental enrichment programme focussing on nutrition, education, and exercise from 3 to 5 years of age was associated with lower schizotypal personality scores at 17 years old [213]. Stress-coping skills interventions on children or adolescents, particularly targeted to vulnerable subgroups, may therefore be a feasible strategy. However, many of the environmental stressors that increase psychosis risk, such as poverty, social isolation, and childhood adversity, are difficult to modify by clinical intervention, and can only be changed through social and political action.

Aerobic exercise (AE) is one promising candidate intervention that exerts both antidepressant and anxiolytic effects, and improves resilience to stress [214]. Patients with schizophrenia and CHR individuals show poorer aerobic fitness than healthy volunteers [215, 216] and FEP with lower physical activity have greater reductions in hippocampal volume compared to FEP with higher physical activity [217]. A longitudinal population study found that self-reported physical activity is lower in youth 9–18 years old that went on to develop a psychotic disorder, with one unit lower on their physical activity index associated with a 26% higher risk for developing psychosis, but not affective or substance use disorders [218]. AE specifically targets the hippocampus, selectively triggering immediate rCBF increases in healthy adults [219], and longer-term training is associated with an increase in hippocampal volume [220], particularly in the anterior hippocampus [221].

AE is effective at reducing symptoms in schizophrenia patients [222]. However, a meta-analysis of four AE studies revealed no significant hippocampal volume increases in schizophrenia or FEP patients [220], though a subsequent study found increased left-CA1 volume in treatment-resistant schizophrenia patients [223]. Plausibly, the benefit of AE may be most marked earlier in the stages of increased vulnerability, coinciding with hippocampal development. In a recent clinical trial, AE improved positive symptoms in individuals at CHR [224]. Moreover, the AE group had stable subiculum volume and increased hippocampal-occipital functional connectivity over the intervention, whereas subiculum volume decreased in the non-AE group. While positive symptom improvements were no longer significant at the 12-month follow-up, these results are encouraging. Large-scale, extended interventions are now needed to determine the effective window for intervention and prevention.

Interestingly, acute exercise induces redox imbalance [225]. However, this temporary imbalance may be essential for triggering repair processes and increasing antioxidant efficiencies, gained through regular training. This double-edged sword of acute versus regular exercise should be considered when implementing aerobic interventions in redox imbalance prone CHR individuals, who are often unreliable reporters of activity [215], and given unsupervised individual interventions are least efficacious [222]. One strategy to mitigate engagement issues may be the use of exercise-oriented videogames [226], which would come at reduced costs, and could be integrated into other computerised interventions such as virtual reality tasks [227].

### Implementing treatments

Psychosis is heterogeneous and multiple aetiologies may contribute to a common pathophysiology. Individual differences in hippocampal dysfunction in psychosis likely arise from different genetic risk, the extent of environmental stressors and protective factors, recreational drug or medication use, as well as the neurodevelopmental timing. Moreover, symptoms overlap with other psychiatric disorders. The aforementioned causes of dysfunction—stress sensitivity, oxidative stress and E/I imbalance—are therefore likely to play a role in the aetiology of other disorders [228, 229], so some treatments may work transdiagnostically. Accordingly, it may be more useful to focus on clusters of overlapping symptoms [230], such as thought disorder, or specific socio-emotional and cognitive deficits. For instance, one study found that thought disorder positively correlated with left amygdala-hippocampus volume loss across major depressive, bipolar, and schizophrenia disorders, irrespective of formal diagnosis [231].

The prospect of prophylactic treatments is tantalising but attempting to correct for imbalances in immature brains should be approached cautiously to avoid unintended consequences on neurodevelopment. Preventive interventions could be provided at different stages of neurodevelopment (Fig. 3), with varying intensity according to stage. The choice of treatment could be tailored to the underlying biology, identified as a deviation from normative markers derived from longitudinal population studies [232] (Fig. 3A). For example, non-specific support could be offered to children and adolescents at increased risk, along with stress-coping skills training, while more specific pharmacological interventions might be appropriate for young adults at CHR, ideally the subgroup of CHR individuals that are most likely to transition to psychosis. The latter might be identified through the assessment of hippocampal and redox dysfunction, with neuroimaging and peripheral blood measures serving as biomarkers [101, 233]. The promise of targeting hippocampal circuit dysfunction lies in reducing the likelihood of transition to psychosis while also addressing underlying transdiagnostic symptoms, such as cognitive deficits.

### Future directions

Longitudinal studies in CHR and other at-risk populations, as well as large community samples, will be critical to mapping maladaptive hippocampal neurodevelopment leading to adverse clinical outcomes. Moreover, hippocampal circuit abnormalities are often first detectable in anterior hippocampal subregions, before dysfunction spreads to surrounding circuits [1]. Consequently, measuring dysfunction within specific hippocampal subregions may be important for understanding the neurodevelopment of psychosis and time-appropriate treatments. Accordingly, it is crucial that any MRI-based segmentation method should also be approached cautiously due to the small size of the hippocampus. A typical voxel size of ~1 mm [3] may be insufficient to segment the hippocampus reliably [234].

Segmentation and functional imaging of the hippocampus will improve as higher field-strength (7T+) scanners become more available. Higher field-strengths also afford the possibility of cortical layer and column analysis [235], allowing for the delineation of hippocampal layering and more specific imbalance localisation. Still, several techniques can boost signal-to-noise ratio (SNR) without new hardware. For instance, reduced-field-of-view imaging around basal ganglia structures rather than whole-brain imaging facilitates sub-1mm [3] voxels without the use of dedicated hardware or invasive imaging contrasts [236].

We have discussed many neuroimaging techniques used to capture early hippocampal abnormalities; these and several emerging technologies, such as optically-pumped magnetoencephalography [237], or chemical exchange saturation transfer [238], may eventually lead to new hippocampal biomarkers for clinical staging in psychosis (Table 1). In addition, other innovative indicators such as maximal oxygen consumption (a measure of aerobic fitness), or gut bacteria diversity—which also impact healthy neurodevelopment and hippocampal processes [239]—could potentially be used as markers of dysfunction and targets for treatment [240, 241].

**Table 1:** Plausible hippocampal anomalies and potential biomarkers in prodromal psychosis and potential tools for measurement.

Expected anomaly	Techniques for detection
Stress-sensitivity	fMRI, galvanic skin response
Abnormal network connectivity	fMRI, EEG, MEG, OP-MEG
Increased metabolism	ASL, fMRI, PET, SPECT
Abnormal hippocampal oscillations	EEG, MEG, OP-MEG
E/I imbalance	^1^H-MRS, fMRI, EEG, MEG, OP-MEG, PET, SPECT
NMDAR hypofunction	PET, SPECT
Redox dysfunction	^1^H-MRS, blood redox markers, VO2 Max
Microbiome disbalance	Stool sample

^*1*^*H-MRS* proton magnetic resonance spectroscopy, *ASL* arterial-spin labelling, *EEG* electroencephalography, *fMRI* functional magnetic resonance imaging, *MEG* magnetoencephalography, O*P-MEG* optically-pumped magnetoencephalography, *PET* positron emission tomography, *SPECT* single-photon emission computed tomography, *VO2 Max* maximum oxygen consumption.

To detect potential markers, the complexity and heterogeneity of psychosis-risk, and how these patterns are divergent from other mental health disorders, it is critical that this multidimensional information is integrated across scales (Fig. 2). This will include not only multi-modal imaging but also the integration of genetic [242] and other neurobiological information [243] in the modelling of the dysfunction. Though the hippocampus is a core hub in the pathology of psychosis, hippocampal abnormalities across scales—genetic, cellular/molecular, whole-brain network dysconnectivity—must be integrated through large-scale collaborative and integrative computational models.

## Conclusions

Improving our understanding of the role of the hippocampus as a central hub of abnormality in the pathophysiology of psychosis may unlock the development of novel treatments and much-needed preventive interventions. Preclinical models indicate that hippocampal changes that occur before the onset of frank psychosis can be reversible, suggesting that clinical interventions at this premorbid stage in humans might be able to reduce the risk of illness onset.
