# Correction: Hippocampal circuit dysfunction in psychosis

**DOI:** 10.1038/s41398-022-02154-y

**Published:** 2022-09-12

**Authors:** Samuel Knight, Robert McCutcheon, Daniella Dwir, Anthony A. Grace, Owen O’Daly, Philip McGuire, Gemma Modinos

**Affiliations:** 1 Department of Psychosis Studies, Institute of Psychiatry, Psychology & Neuroscience, King’s College London, London, UK; 2 Center for Psychiatric Neuroscience, Department of Psychiatry, Lausanne University Hospital (CHUV), Lausanne, Switzerland; 3 Departments of Neuroscience, Psychiatry and Psychology, University of Pittsburgh, Pittsburgh, PA USA; 4 Department of Neuroimaging, Institute of Psychiatry, Psychology and Neuroscience, King’s College London, London, UK; 5 NIHR Maudsley Biomedical Research Centre, London, UK; 6 MRC Centre for Neurodevelopmental Disorders, King’s College London, London, UK

**Keywords:** Schizophrenia, Biomarkers, Clinical pharmacology

Correction to: *Translational Psychiatry* 10.1038/s41398-022-02115-5, published online 25 August 2022

The original version of this article unfortunately contained an error in the references. Reference 91 refers to:

Kiemes A, Gomes FV, Cash D, Uliana DL, Simmons C, Singh N, et al. GABAA and NMDA receptor density alterations and their behavioral correlates in the gestational methylazoxymethanol acetate model for schizophrenia. Neuropsychopharmacology. 2022;47:687–95. However, it should refer to: Kiemes A, Serrano Navacerrada ME, Kim E, Randall K, Simmons C, Rojo Gonzalez L et al. Erbb4 deletion from fast-spiking interneurons causes psychosis-relevant neuroimaging phenotypes. bioRxiv. 2022: 2022.2003.2007.483347. The original article has been corrected accordingly.

